# Gastrointestinal Beriberi Secondary to Intermittent Hemodialysis With Rapid Improvement Following Thiamine Administration

**DOI:** 10.1155/crgm/3195006

**Published:** 2026-06-08

**Authors:** Benjamin Dale Day, Jacelyn Emily Peabody Lever, Kathryn Vickers, Robert Centor

**Affiliations:** ^1^ Department of Internal Medicine, Tinsley Harrison Internal Medicine Residency Program, University of Alabama at Birmingham, Birmingham, Alabama, USA, uab.edu; ^2^ Department of Anesthesiology and Perioperative Medicine, Anesthesiology Residency Program, University of Alabama at Birmingham, Birmingham, Alabama, USA, uab.edu

## Abstract

Thiamine deficiency is classically linked to malnutrition and chronic alcohol use, but emerging evidence indicates that patients on intermittent hemodialysis (iHD) are also at significant risk. While cardiovascular and neurologic syndromes are well characterized, gastrointestinal (GI) beriberi remains an underrecognized presentation. We describe a 67‐year‐old man on iHD with chronic abdominal pain, nausea, vomiting, and anorexia refractory to conventional therapy. Workup revealed elevated lactate and a low plasma thiamine level prior to supplementation. Symptoms and elevated lactate resolved rapidly following intravenous thiamine. This case underscores GI beriberi as a treatable cause of chronic GI symptoms in dialysis patients.

## 1. Introduction

Thiamine (vitamin B_1_) is an essential cofactor for oxidative metabolism, and deficiency disrupts carbohydrate processing, resulting in lactate accumulation and multisystem dysfunction. Classic manifestations include cardiovascular (wet beriberi), neurologic (dry beriberi, Wernicke’s encephalopathy), or mixed syndromes. However, a distinct presentation—gastrointestinal (GI) beriberi—has been described, characterized by nausea, vomiting, anorexia, and abdominal pain [1–3].

Patients undergoing intermittent hemodialysis (iHD) are particularly susceptible to thiamine deficiency due to dialytic clearance, with plasma levels declining by up to 45% after a single session [4]. Several reports have described classic beriberi syndromes in this population, often preceded by GI symptoms [5–9]. Nausea, vomiting, abdominal pain, and anorexia are also common among dialysis patients and are frequently attributed to conditions including uremic gastropathy, medication‐related adverse effects, gastroesophageal reflux disease (GERD), and functional bowel disorders (FBD) [10, 11]. Because thiamine deficiency can present with similar nonspecific symptoms, GI beriberi may represent an underrecognized and potentially reversible contributor to chronic GI complaints in patients receiving dialysis.

This report describes an iHD patient with chronic, refractory GI symptoms and newly elevated lactate who experienced complete resolution after thiamine repletion, highlighting diagnostic considerations and clinical implications.

## 2. Case Report

A 67‐year‐old male with chronic kidney disease (CKD) on iHD (monday/wednesday/friday via left upper extremity fistula) for one year, anemia of CKD, bipolar I disorder, hypertension, and type II diabetes presented to the emergency department (ED) with worsening chronic abdominal pain and vomiting and was subsequently admitted to the internal medicine service. GI symptoms began 1‐2 weeks after initiation of dialysis and progressively worsened over the subsequent 12 months. He described dull, nonradiating, poorly localized abdominal pain associated with nausea, vomiting, and food aversion. Initially, intermittent symptoms progressively worsened over the preceding 2 months, ultimately becoming constant with three to five episodes of nonbloody, nonbilious vomiting daily. Notably, symptoms were not temporally associated with dialysis sessions and did not predictably worsen prior to or improve following dialysis treatments. The pain worsened with oral intake, leading to significant anorexia and a 16‐pound weight loss over the prior year. He denied bowel changes, GI bleeding, confusion, neurologic deficits, or symptoms indicative of heart failure. He had tolerated his last iHD session 24 h earlier without complication. There was no personal history of alcohol, illicit substance, or tobacco use. At the time of dialysis initiation, his diet consisted of three balanced meals daily with regular protein intake. However, oral intake progressively declined following the onset of GI symptoms and worsened substantially over the preceding 2 months because of symptom progression. His prescribed medications included rosuvastatin, amlodipine, aripiprazole, insulin glargine, iron supplementation, weekly darbepoetin, and a daily renal multivitamin, though he acknowledged intermittent adherence to outpatient medications, including vitamin supplementation.

Although he remained adherent to scheduled dialysis treatments, he frequently missed outpatient nephrology follow‐up and had not established care with a primary care physician despite repeated scheduling efforts. Consequently, much of his evaluation and care occurred through recurrent ED encounters. Over the preceding 10 months, he had presented to the ED 10 times for similar symptoms, including 5 visits within the prior 5 weeks, several of which resulted in hospital admission.

ED and inpatient evaluations included multiple abdominal CT scans and ultrasounds without acute abnormalities. Gastroenterology consultations resulted in esophagogastroduodenoscopy demonstrating mild gastritis and colonoscopy notable only for a solitary 6‐mm tubular adenoma that was removed, and a normal gastric emptying study. Broad laboratory evaluation across multiple encounters was unrevealing, including normal lactate, liver function, thyroid studies, celiac serologies, lipase, cardiac biomarkers, vitamin B12, folate, and inflammatory markers. Despite treatment with pantoprazole, dicyclomine, antiemetics, and other symptomatic therapies, symptoms persisted. As the unrevealing diagnostic evaluation and underlying psychiatric history increasingly raised concern for functional and psychiatric etiologies, subsequent encounters became more focused on symptom management rather than further diagnostic investigation.

### 2.1. Physical Examination

He appeared uncomfortable but in no distress. Blood pressure is 134/78, and oxygen saturation is 100% on room air. Vitals were otherwise normal. The abdomen was soft with diffuse tenderness and active bowel sounds, without guarding or rebound. Cardiopulmonary examination revealed clear lung fields without crackles and no jugular venous distension, murmurs, or peripheral edema. Neurologic exam revealed appropriate cognition, normal strength, and sensation throughout.

### 2.2. Laboratory Findings

Hemoglobin was 8.0 g/dL (13.5–17.5 g/dL), creatinine was 8.7 mg/dL (0.7–1.2 mg/dL), blood urea nitrogen was 25 mg/dL (8–24 mg/dL), HCO_3_ was 23 mEq/L (22–28 mEq/L); electrolytes, liver function, thyroid function, B_12_, folate, and iron studies were normal. Lactic acid was 3.0 mmol/L (0.5–2.2 mmol/L).

This was the first documented lactate elevation during one of the patient’s symptomatic episodes and served as an important diagnostic clue in the broader clinical context. Although the elevation was mild and nonspecific, the patient was mildly hypertensive, not hypoxic, nontoxic appearing, and without clinical evidence of hypoperfusion or organ ischemia, making common causes of type A lactic acidosis less likely. Given the chronicity of symptoms, dialysis‐associated risk factors for thiamine deficiency, and previously unrevealing structural evaluations, differential diagnoses included mesenteric ischemia, GI beriberi, diabetic autonomic neuropathy, and medication adverse effects.

A plasma thiamine level was obtained prior to empiric administration of 200 mg intravenous thiamine. The pretreatment thiamine level later returned at 60 nmol/L (reference range: 78–185 nmol/L). Within 12 h, symptoms resolved completely, and he tolerated solid food for the first time in weeks. Repeat lactate decreased to 2.0 mmol/L. He received a second 200 mg intravenous dose and was transitioned to oral thiamine 100 mg daily.

The patient remained symptom‐free throughout hospitalization and at discharge 48 h later. Repeat thiamine testing after supplementation demonstrated normalization to 218 nmol/L. Over 5 months of follow‐up, there were no further ED visits for GI complaints.

## 3. Discussion

This case demonstrates GI beriberi as a reversible manifestation of thiamine deficiency in an iHD patient. Diagnostic confidence was supported by chronic GI symptoms, elevated lactate, low pretreatment thiamine level, rapid and sustained clinical response, and exclusion of alternative etiologies. The patient’s presentation is biologically consistent with thiamine’s central role in oxidative metabolism and GI neuromuscular function, particularly given the increased risk of deficiency among patients receiving iHD.

Thiamine deficiency impairs aerobic glucose metabolism, promoting lactate accumulation and cellular energy depletion that may disrupt GI smooth muscle and enteric nervous system function, contributing to nausea, vomiting, and abdominal pain [12]. Patients receiving iHD are particularly susceptible because thiamine is water soluble, has limited body stores, and is readily cleared during dialysis sessions. Reduced nutritional intake and inconsistent adherence to supplementation may further increase risk, and routine supplementation may not fully compensate for ongoing dialytic losses in some patients.

Diagnostic criteria for GI beriberi remain undefined, but proposed features include (1) chronic or recurrent GI symptoms refractory to standard therapy, (2) objective or functional evidence of thiamine deficiency, and (3) rapid resolution with thiamine replacement [1]. This patient fulfilled all three criteria, with normalization of lactate strengthening diagnostic confidence. Although whole blood thiamine diphosphate measurement is preferred because it better reflects intracellular stores, only plasma thiamine testing was readily available at our institution. Despite this limitation, the patient demonstrated a low pretreatment thiamine level along with marked clinical improvement following supplementation.

Alternative diagnoses including uremic symptoms, gastroparesis, ischemia, inflammatory disease, thyroid dysfunction, and medication adverse effects were considered but effectively excluded through longitudinal evaluation. Multiple prior abdominal imaging studies, normal gastric emptying, and largely unrevealing endoscopic evaluation made structural, inflammatory, and motility‐related etiologies unlikely. Laboratory trends demonstrated stable dialysis‐associated metabolic parameters without evidence of worsening uremia correlating with symptom severity. Medication review revealed no clear offending agent, while rapid symptom and lactate improvement following thiamine administration made ischemia unlikely and further supported GI beriberi as the primary diagnosis.

There are no standardized dosing guidelines for thiamine replacement in iHD patients. Most treatment recommendations for beriberi have used 100–500 mg IV daily for 1–3 days, followed by oral maintenance [13]. Given enhanced dialytic clearance, higher or more frequent dosing strategies may be necessary, though this remains an area requiring further study. Our patient improved rapidly with two 200 mg IV doses.

The prolonged diagnostic course in this case likely reflects several factors that contribute to delayed recognition of GI beriberi in clinical practice. Early symptoms substantially overlapped with more common GI disorders. Prior to the eventual lactate elevation, there were few objective findings specifically suggestive of thiamine deficiency, and the patient underwent an extensive and otherwise appropriate evaluation that repeatedly excluded more common etiologies. Additionally, fragmented longitudinal care, reliance on emergency department encounters, and increasing attribution of symptoms to functional and psychiatric causes may have further limited consideration of nutritional deficiencies. This case highlights the importance of maintaining awareness of GI beriberi in at‐risk populations, particularly given the safety, low cost, and potential diagnostic value of empiric thiamine supplementation, which in this patient may have prevented prolonged morbidity and recurrent healthcare utilization had it been considered earlier.

## 4. Conclusion

GI beriberi is a reversible but underrecognized complication of thiamine deficiency in patients undergoing iHD. Chronic GI symptoms refractory to standard management should prompt consideration of this diagnosis in at‐risk patients, particularly when accompanied by elevated lactate, though normal lactate levels do not exclude the condition. Given thiamine’s safety and low cost, empiric supplementation is both reasonable and potentially diagnostic. Early recognition and treatment may prevent prolonged morbidity, unnecessary diagnostic testing, and recurrent healthcare utilization.

## Funding

No funding was received for this manuscript.

## Conflicts of Interest

The authors declare no conflicts of interest.

## Data Availability

Data sharing is not applicable to this article as no datasets were generated or analyzed during the current study.
